# Retinal toxicity associated with chronic exposure to hydroxychloroquine and its ocular screening. Review

**Published:** 2014-09-25

**Authors:** A Geamănu (Pancă), A Popa-Cherecheanu, B Marinescu, CD Geamănu, LM Voinea

**Affiliations:** * Ophthalmology Department, "Carol Davila" University of Medicine and Pharmacy, Bucharest; ** "Victor Babeș"’ National Institute of Pathology, Bucharest; *** "Foisor" Traumatology and Orthopedics Clinical Hospital, Bucharest

**Keywords:** hydroxychloroquine sulfate, bull’s eye maculopathy, screening tool

## Abstract

Abstract

Hydroxychloroquine sulfate (HCQ, Plaquenil) is an analogue of chloroquine (CQ), an antimalarial agent, used for the treatment of systemic lupus erythematosus, rheumatoid arthritis and other autoimmune disorders. Its use has been associated with severe retinal toxicity, requiring a discontinuation of therapy. Because it presents potential secondary effects including irreversible maculopathy, knowledge of incidence, risk factors, drug toxicity and protocol screening of the patients it represents important data for the ophthalmologists.

Thus, it is imperative that rheumatologists, medical internists and ophthalmologists are aware of the toxicity from hydroxychloroquine they should also be careful to minimize its occurrence and effects.

## Introduction

Hydroxychloroquine sulfate is an antimalarial agent used for the treatment of systemic lupus erythematosus, rheumatoid arthritis and other autoimmune, inflammatory and dermatologic conditions, with less toxic effects than chloroquine.

 The most concerning side effect is retinal toxicity and thus, it is vital for ophthalmologists to know the incidence, risk factors, drug toxicity and protocol screening of the patients who take this medication.

 Incidence and Risk Factors 

 The incidence of toxic retinopathy varies from 0%-4%. A prospective study of Mavrikakis I et al, published in Ophthalmology, in 2003, on 526 patients over 15 years old found the incidence to be 0.38% [**1**].

 A new study of Wolke F and Marmor MF, published in Arthritis Care & Research, in 2010, on approximately 4000 unrelated patients with rheumatoid disorders in treatment with hydroxychloroquine found a higher prevalence of HCQ toxicity (6.8/1000 users), but the prevalence was dependent on the duration of use (cumulative dose). The prevalence was only a few per 1000 within the first 5 years of use and increased sharply after 5 to 7 years to approximately 1% [**2**]. A cumulative dose of 1000g HCQ is reached in 7 years with a typical daily dose of 400 mg.

 In the same study, toxicity was not related to age, weight or daily dose but was related to duration of use and increased rapidly after 5 to 7 years; thus, it is important to be aware of the risk factors and use more effective protocols of screening on all the patients who exceed 5 years of exposure.

**Table 1:** Criteria of low- and high- risk patients for the development of hydroxychloroquine maculopathy [**3**]

HCQ therapy	Low risk	High risk
Daily dose	< 6.5 mg/kg ideal body weight for short individuals 200- 400mg/day	> 6.5 mg/kg ideal body weight for short individuals > 400mg/day
Duration of use	<5 yrs	> 5 yrs
Cumulative dose	< 1000g( total)	> 1000g( total)
Kidney / liver dysfunction	-	+
Retinal disease or maculopathy*	-	+
Age ( with no cut point specified)		elderly
*Patients with underlying retinal disease may be at high risk for toxicity and it is considered by many to be a contraindication for HCQ use because it masks the early signs of toxicity and it makes screening less effective or impossible.

 Although, the American Academy of Ophthalmology indicates that the cumulative dose is the most important risk for toxicity, an editorial from 2012, published in the Canadian Journal of Ophthalmology suggested that the daily dose or dose/kg was at least as important. 

 The incidence of retinopathy has significantly declined in recent years because rheumatologists do not routinely prescribe 400mg/day HCQ but are appropriately treating patients based on the ideal body weight (IBW) [**4**].

 IBW depends on weight (kg) and height (inch) and is different for males and females. For example:

 - for males, IBW= 51.65kg + 1.85kg/inch greater than 5 feet (1.52m); 1 inch = 2.54cm

 - for females, IBW = 48.67kg + 1.65kg/inch greater than 5 feet (1.52m) [**5**].

 In practice, most subjects receive the "typical" dose of 400mg (2 tables) regardless of weight and we should be more cautious with individuals of short stature to whom a daily dose of 400mg is too high and therefore the risk for retinal toxicity by overdose is increased. Thus, for subjects who are of short stature, daily doses must be calculated on the basis of ideal body weight.

 Kidney or liver disorders can decrease the effective rate of drug removal which in effect increases the blood level because the clearance of hydroxychloroquine is made by both the kidney and the liver.

 Elderly patients may be at high risk since the assessment of toxicity is more difficult because with age, the diffuse loss of fundus pigmentation makes the bull’s eye maculopathy hard to recognize.

 Among the risk factors associated with maculopathy the most important were the cumulative and daily doses, although we found conflicting results in different researches. 

 A new study published in Reumatologia Clinica, in 2013, found a positive association between retinal toxicity and hypertension [**6**]. The induction of oxidative stress and endothelial dysfunction in case of hypertension [**7**] could explain this speculation, as it was demonstrated that the two processes acted as promoters of vascular damage and progressive atherosclerotic with further thrombotic complications in the vascular walls. 

 Mechanism of action 

 The mechanism of hydroxychloroquine toxicity is not completely understood. Although the earliest changes appear in the cytoplasm of ganglion cells and photoreceptors, with later involvement of the retinal pigment epithelium (RPE) where the drug binds to melanin [**8**], it may adversely influence the metabolism of the retinal cells and may lead to the slow and chronic toxic effects.

 Another hypothesis based on macular localization of the toxicity is that the light absorption or cone metabolism may play a role in its effect [**9**].

 Clearance of the HCQ from blood and urine can take months to years after drug cessation and visual function may continue to deteriorate slowly even after the therapy was stopped [**1**].

 Hydroxychloroquine Retinopathy

 Chloroquine retinopathy was described for the first time in the early ’60s. Hydroxychloroquine and chloroquine work in a similar way as antimalarial medication with different therapeutic and toxic doses and produce a similar pattern of retinopathy.

 HCQ appears to be considerably less toxic to the retina than CQ, possibly because chloroquine passes the blood – retinal barrier easier [**10**].

 Symptoms 

 In the early stages of HCQ retinal toxicity, most of the patients could be asymptomatic. When the first symptoms start to appear their complaints are the following: trouble with reading, diminished color vision, fine visual alteration due to the central or paracentral scotoma.

 Signs

 Biomicroscopy eye exam (slip lamp examination). Quinolones can precipitate in the corneal epithelium in a diffuse punctate or whorl-like pattern which sometimes can result in visual haloes [**11**]. This sign is less common with HCQ than CQ and its effect is reversible when the therapy is stopped [**12**].

 Visual Field examination. The earliest scotomas are subtle, usually within 10 grades of fixation, and are more common superiorly than inferiorly to fixation [**13**]. In time, the scotomas enlarge, multiply and involve fixation, which reduces visual acuity.

 The fundus examination (ophthalmoscopy) can remain completely normal even after the central scotoma’s development. The earliest signs of toxicity are the fine pigment stippling of the macula, some irregular pigmentation changes and loss of the foveal light reflex, sometimes referred to as premaculopathy [**11**].

 In time, this may progress and the irregular central pigmentation may become surrounded by the annular zone of depigmentation of the retinal pigment epithelium, preponderantly inferior to the fovea. This pattern appears in an advanced stage, called bull’s eye maculopathy, with a corresponding perifoveal visual field defect.

 When allowed to advance, HCQ retinal toxicity produces a generalized atrophy of the retinal pigment epithelium and leads to the loss of up to three visual functions: visual acuity, peripheral visual field and night vision [**3**]. In this stage, it is hard to differentiate it from the retinitis pigmentosa [**14**].

**Table 2:** Where can we find the bull’s eye maculopathy?

- HQ/CQ retinal toxicity - Age related macular degeneration - dry stage - Benign concentric annular dystrophy - Central areolar choroidal dystrophy - Chronic macular hole - Cone and cone-rod dystrophies - Stargardt disease

**Fig. 1:**
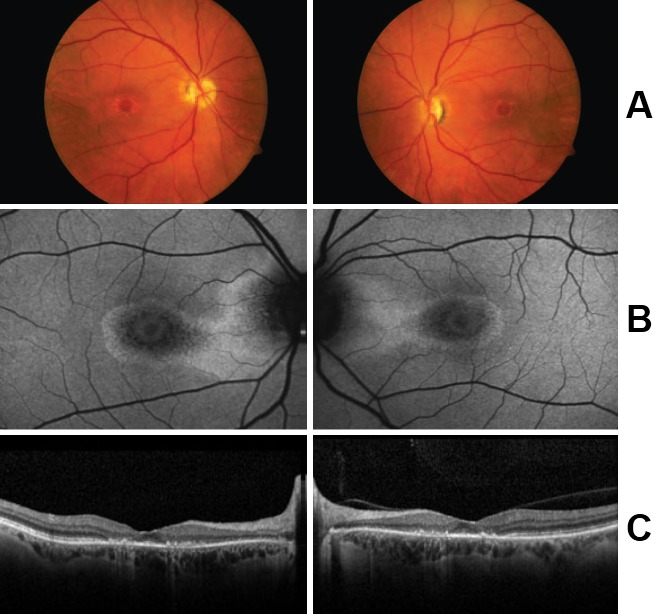
[**14**] Bull’s eye maculopathy. A 55-year-old female who had been taking hydroxychloroquine for 10 years before the onset of symptoms. (A) Color fundus photos showing bull’s-eye maculopathy. (B) Fundus autofluorescence with central mottled hypo autofluorescence with the surrounding rim of hyper autofluorescence. (C) SD-OCT shows marked parafoveal thinning of the retina, especially of the outer photoreceptor layers.

 Screening of the patients treated with HCQ - actual practice

 New recommendations for the screening of HCQ/CQ maculopathy were provided by The American Academy of Ophthalmology in 2011, which stressed the necessity for the acknowledgement of the prevalence of toxicity, the improvement of the assessment tools of the patients and the earlier detection of secondary retinal effects [**3**].

 No treatment has yet been discovered for this disorder, so it is imperative that a good collaboration between the patient and the multidisciplinary physicians’ team (medical internist, rheumatologist, and ophthalmologist) exists in order to minimize the toxic damage and become aware of their best practices.

 A baseline examination before starting the HCQ therapy is considered as a reference point, due to the possibility of detecting any maculopathy (for example, age related macular degeneration), which might be a contraindication to the drug use.

 The annual screening should begin no sooner than 5 years after starting the therapy and even earlier in cases which have associated risk factors (**Table 1**).

 Although there are no evidence-based guidelines on screening, some researchers prefer to see the patients every 2 years after the initiation of the therapy, so that they will not be lost for the follow-up [**4**].

 In practice, an ophthalmological exam (fundus exam + central perimetry 10-2) and at least one objective test it is recommended, if possible [**15**].

**Table 3:** Baseline examination for patients treated with HCQ

Subjective tests	Objective tests
Visual acuity for distance/reading Slit lamp examination (cornea) Fundus examination Automated central perimetry 10-2 (Humphrey visual field 10-2) Fundus photography- optional , if exist pretreatment macular changes
Fundus autofluorescence or mf ERG or OCT- macula

 An ophthalmological exam before the initiation of the drug therapy is necessary for the actual ocular assessment. The fundus examination has an important role for the detection of any macular changes but, it is not considered a sensitive screening tool because it does not detect the early stage of the retinal toxicity [**16**].

 The retinopathy, funduscopically visible bull’s eye maculopathy, indicates that severe toxicity has determined irreversible degeneration of the retinal pigment epithelium and since it is already a late stage, a screening test is not necessary anymore [**17**].

 Standard automated perimetry 10-2 testing with white spot can be a sensitive test for the early stages of toxicity, it must include pattern deviation plots [**18**] and its effectiveness which depends on the patient’s cooperation and the user’ experience is critical. Red testing can be more sensitive than the white one, but is less specific [**19**].

 Moderate focal loss of retinal sensitivity must be recognized and taken into consideration through a serious evaluation, repeating the visual field exam and being completed by the objective test to confirm the maculopathy. In an advanced and tardive stage, the visual field shows a typical perifoveal scotoma [**15**].

 In an editorial published in 2013, Marmor MF recommended at least 2 methods (1 objective) to confirm toxicity before stopping HCQ, in order to be sure that those field defects are not a subjective variation.

 Fundus autofluorescence (FAF) indicates marked changes of macular toxicity in the mild and severe cases, represented by the central mottled hypo autofluorescence with surrounding rim of hyper autofluorescence, due to the retinal pigment epithelium (RPE) loss [**20**]. FAF is a sensitive indicator of the RPE degeneration caused by the toxic progression.

 The value of the mfERG in retinal toxicity is related to the stage of disorder and it is a useful exam to document localized paracentral ERG depression in the early cases [**21**] or to confirm the bull’s eye maculopathy [**22**]. The mfERG should follow the International Society for Clinical Electrophysiology of Vision’s principles and it should look for a relative signal of weakness in the parafoveal rings [**23**]. There is evidence that the mfERG may be more sensitive to early paracentral functional loss than the white 10-2 field [**17**].

 The SD-OCT (high resolution OCT) can show a localized thinning of the retinal layers in the parafoveal region and confirm the toxicity in early stages, before the symptomatic visual field loss [**24,25**].

 Early HCQ retinopathy defects were found to present a loss of the perifoveal photoreceptor inner segment/outer segment (IS/OS) junction with intact outer retina directly under the fovea, creating the „flying saucer" sign, sometimes even before the ophthalmoscopic fundus’ changes are apparent [**26**].

 Recent studies have shown that the SD-OCT is a sensitive examination tool, detecting the disruption of the perifoveal photoreceptor IS/OS junction even before the retinal pigment epithelium’s abnormalities [**27**].

 Amsler grid testing, color vision testing, fluorescein angiography, full-field electroretinogram and electrooculogram are no longer recommended [**3**].

 The new objective tests are more sensitive as they make a fine assessment of the retinal changes even from early stages when there are no visible signs but abnormalities of the visual field and changes of the OCT or mf-ERG are observed.

 Patients should be aware of the risk of toxicity and of the rationale for screening in order to detect early changes and minimize visual loss, not necessary to prevent it [**3**].

 Retinal toxicity is a very rare side effect of the hydroxychloroquine therapy, but when it has occurred, vision loss may be permanent and may progress even years after the cessation of medication. Patients would be examined every 3 months, then annually, until they are stabled.

 The drugs should be discontinued when toxicity is recognized or strongly suspected but this is a decision to be made in conjunction with the patient – the rheumatologist, responsible with hydroxychloroquine administration and - the ophthalmologist, responsible with visual screening.
